# The mediating effect of subjective well-being in the relationship between social support and professional commitment among mainland Chinese kindergarten teachers

**DOI:** 10.3389/fpsyg.2022.1011855

**Published:** 2022-09-27

**Authors:** Shujuan Chen, Yun Luo, Zheyuan Mai, Xiaojing Chen, Taoyu Shen

**Affiliations:** ^1^School of Education, Zhaoqing University, Zhaoqing, China; ^2^Department of Social and Behavioural Sciences, City University of Hong Kong, Kowloon, Hong Kong SAR, China

**Keywords:** kindergarten, professional commitment, subjective well-being, social support, positive affect, life satisfaction

## Abstract

Kindergarten teachers’ professional commitment affects their emotional input and turnover intention, and it is affected by the spiritual and material factors of teachers’ families, kindergartens, and society. Therefore, this study aimed to investigate the mediating effect of the dimensions of subjective well-being in the relationship between social support and professional commitment. The study is grounded in human ecology theory and social exchange theory. We surveyed 778 kindergarten teachers from different educational systems in Guangdong Province in China. We used the “Appreciative Social Support” and “Subjective Well-being” Scales and the “Professional Commitment Questionnaire.” The results showed that the four variables of social support, positive affect, life satisfaction, and professional commitment of kindergarten teachers were significantly and positively correlated with each other: social support positively predicted positive affect and life satisfaction, positive affect and life satisfaction positively predicted professional commitment, and social support indirectly influenced professional commitment through the parallel and chain path of positive affect and life satisfaction. This represents a compound multiple mediating effects on professional commitment.

## Introduction

Kindergarten teachers (KTs) have the important responsibility of fostering children’s wisdom and shaping their personalities. Preschool children are often vulnerable and poorly socialized, which makes the work of teachers complicated and the responsibility, heavy (Zhang, 2004; Lu and Wang, 2006; Hu, 2020). Additionally, in China, there is a shortage of work resources, and the working conditions are relatively poor (Fan and Li, 2018); KTs are one of the lowest-paid professional groups in the teaching force (Nelson, 2001; Hu, 2020). Heavy workload and low salary are important predictors of Chinese teachers’ turnover intention (Liu and Onwuegbuzie, 2012; Hu, 2020). High rates of resignations and burnout of teachers have become obstacles to the development of preschool education in China (Wang et al., 2015; Zhou et al., 2020), and this has become a worldwide problem (Hong, 2010; Wells, 2015). Most KTs stay in the front line of early childhood education throughout their lives; they are loyal to their duties, care for young children, love their jobs, have deep feelings for young children, and show a high degree of recognition and emotional dependence on their profession. High levels of professional commitment (PC)—the individual teacher’s internal “psychological contract”—and internalization of social norms result in reluctance to change their profession (Tang et al., 2015). What forces support them in maintaining their PC? Is it the spiritual satisfaction from the profession or the material satisfaction from the salary, or the support and affirmation from family, kindergarten children, or friends? Or is it the personal happiness that comes from the realization of self-worth in the profession? Various interacting factors affect the PC of KTs; thus, it is beneficial to improve PC by exploring the influence of each factor.

## Literature review

### Social support and professional commitment

PC is defined as the “practitioner’s emotion, or attitude about their positions” (Rusbult and Farrell, 1983; Blau, 1985; Lee et al., 2000), “the individual’s motivation to continue in the job” (Hall, 1968), “the psychological connection the practitioner establishes with the positions” (Vandenberg and Scarpello, 1994), and “the individual’s identification, enjoyment, and engagement with their positions” (Aranya et al., 1981; Meyer et al., 1993; Deng et al., 2019; Wang, 2019). These perspectives emphasize a unidimensional feature of PC, focusing on the practitioners’ emotions and motivations toward the occupation, rather than the full picture of PC. Meyer et al. (2012) define it from a multidimensional perspective; they define PC as the degree to which individuals internalize their professions through their emotions, commitment, and social norms, such that they are unwilling to change their occupations. The definition includes three dimensions: affective commitment, continuance commitment, and normative commitment. A teacher’s PC is the manifestation of occupational commitment in the field of education and refers to an individual teacher’s feelings toward education and motivation to choose to continue in the profession (Rusbult and Farrell, 1983). It can also refer to teachers’ occupational identity (Meyer et al., 1993), sense of belonging, work commitment, and willingness to continue in the teaching profession (Hu and Li, 2018). KTs’ PC refers to the behaviors and attitudes that are formed based on the internalization of norms about whether they can maintain loyalty to their profession (Chen and He, 2015; Liu, 2018), It is the combination of KTs’ emotional input, loyalty, and willingness to stick to their posts.

Human ecology is a science that studies the relationship between individuals and the environment. Researchers analyze the relationship between individual careers and the environment from the human ecological perspective, and research has found that retention decisions are the result of the interaction between decision-makers and the work environment (Danes and Rettig, 1995). Moreover, social support—as one of the environmental factors—is closely related to PC and the intention to stay (Pomaki et al., 2010; Tang et al., 2015). Conversely, organizational commitment is negatively related to the propensity to leave (Chen and Li, 2009). Principal and colleague support also has a direct effect on teachers’ PC (Kusum and Billingsley, 1998; Singh and Billingsley, 1998), and family support is positively related to the individual achievements of educators (Bataineh, 2009). Teachers’ initiative, participation in decision-making, information feedback, cooperation, learning opportunities, available resources, and other factors influence their PC (Firestone and Pennell, 1993). KTs’ support from the organization and society is positively related to job performance (McGinty et al., 2008); support from family/friends is significantly and negatively related to teachers’ turnover intention (Zhou et al., 2020), and KTs’ social support is significantly and positively related to PC (Tang et al., 2015; Shi, 2017; Xu et al., 2017). Work environment, job satisfaction, and recognition by others are positively related to the level of professional commitment of novice KTs (Feng, 2020), that is, if teachers receive more social support, they show higher PC and retention (Guo et al., 2021).

### Social support and subjective well-being

Sarason (1981) argues that social support “is the subjective perception of interaction or the objective existence of interaction;” Wills (1991) argues that social support is “the individual’s perception of being cared for, respected, and part of a mutually supportive social network.” This is the understanding of social support from the perspective of social interaction. In this sense, social support is a two-way activity, which is the act of offering mutual help and support when individuals encounter difficulties. Such social support comes from social or community organizations (Taylor, 2011), superiors (Bruce and Blackburn, 1992; Vroom, 2008), peers (Chiaburu and Harrison, 2008), family, friends, or other groups (Sarason et al., 1990). Social support can be divided into subjective and objective forms (Barrera, 1986; Vangelisti, 2009). Subjective social support, that is, moral support, refers to when individuals feel their importance and value in social relationships, such as being valued, respected, and liked by others. Objective social support, that is, material support, refers to individuals receiving financial help and material support from society or organizations. Social support comprises care for individuals in need by the provision of practical and material support. However, it is also a potential resource that can be used when individuals are managing stress and solving problems; in such a case, it is referred to as moral support. KTs’ social support is often derived from moral and material care, comfort, and help from family, relatives, friends, classmates, leaders, colleagues, and other members of society (Qu, 2019). Zhang (2012) finds that Chinese KTs’ had moderate-to-high levels of social support, with significant differences according to marital status, age, teaching experience, class size, and monthly salary. Social support was significantly correlated with subjective well-being (SWB; Zeng, 2009; Song and Fan, 2013; Lu and Yang, 2014; Pu, 2014; Zhao, 2016). The more subjective support available for KTs, the higher the level of SWB (Zeng, 2009), which, in turn, had a positive effect on support utilization (Li, 2007).

### Subjective well-being and professional commitment

SWB is a holistic assessment of an individual’s quality of life—based on personal criteria—and consists of two main components: life satisfaction (LS) and affective index (Diener, 1984). LS belongs to the cognitive aspect of SWB, while the affective index belongs to emotional experience, that is, experiencing positive emotions and a lack of negative emotions. This model of psychological well-being comes from the contributions of Diener (1984) and Diener et al (1999). Factors affecting teachers’ SWB include individual factors, such as personality (Ni et al., 2006; Chen et al., 2012; Mao and Xie, 2013), individual teaching motivation (Li et al., 2010), and individual professional psychology (Luo et al., 2006; Zuo and Xi, 2008; Cui, 2010; Li, 2010; Chen et al., 2012; Zhou, 2014). It also includes social factors such as life events, quality of survival, coping styles, interpersonal psychology, academic environment, marital stress, and socioeconomic status (Cui, 2016; Luo and Wang, 2016; Lei and Li, 2018). The influence of teachers’ SWB on their work attitudes and behaviors is mainly reflected in work engagement, work stress, burnout, professional identity, and PC (Wang, 2014; Pi, 2018; Li, 2019; Wu, 2019; Wang and Wang, 2020; Peng, 2021). The higher teachers’ SWB, the higher their work engagement, professional identity, and PC (Wang, 2014; Pi, 2018; Peng, 2021).

### Positive affect, life satisfaction, and subjective well-being

According to social exchange theory (Gouldner, 1960; Blau, 1964), social support predicts and promotes employees’ job satisfaction (Ferguson et al., 2012; Zhang et al., 2015). When employees receive support from organizational leaders and colleagues, they enjoy their jobs and are more loyal to the organization. Conversely, when employees do not receive support, it leads to job dissatisfaction and reduced loyalty to the organization (Lambert et al., 2016). Different dimensions of social support can predict SWB (Xu, 2011), and social support affects PA (Zhao, 2016). PA has the highest correlation with instrumental support; LS has the highest correlation with emotional support, and PA influences LS. The more emotional support KTs receive, the higher their LS and SWB, and the lower their negative affect (Jiang, 2020). KTs’ quality of work life is related to PC (Daud, 2010). Job satisfaction plays a mediating role, with the work environment and recognition from others indirectly influencing PC through job satisfaction (Feng, 2020). KTs will experience higher job satisfaction and be more loyal to the organization when they have good relationships with children, managers, and colleagues, and have opportunities to grow (Daud, 2010). KTs’ social support affects their job satisfaction directly, and indirectly through the single-path mediating role of job engagement and the chain-mediating role of self-efficacy and job engagement (Wu et al., 2020). Concerning the role of the PA aspect of social support in SWB, instrumental support stimulates PA, affective support influences LS, and PA influences LS, which enhances personal SWB. Moreover, KTs with high SWB generate high PC. There are reasons to believe that PA and LS mediate between social support and the PC of KTs, and this mediation occurs as a compound multiple mediating effects (Liu and Ling, 2009).

Thus, we propose the following research hypotheses:

*H1:* PA mediates between KTs’ social support and PC.

*H2:* LS mediates between KTs’ social support and PC.

*H3:* PA and LS play a multiple mediating role in the relationship between KTs’ social support and PC.

We construct a compound multiple mediating effect theoretical model to explore the mediating roles of PA and LS between social support and the PC of KTs to provide a theoretical basis for their PC and management.

**Figure 1:**
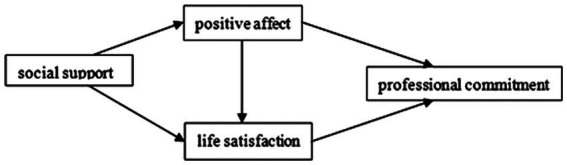
The posited model.

The complete hypothesized model is presented in Figure 1.

## Methods

### Participants and data collection

Participants comprised 778 KTs from all over Guangdong Province, which is a developed area in China. Their ages range from 18 to 55 years (*M* = 31.75, SD = 8.26), and their years of experience range from 0 to 38 years (*M* = 9.75, SD = 8.37).

### Measures

#### Social support

This study assessed social support using the Chinese version of the Perceived Social Support Scale (PSSS). The PSSS was originally developed by Zimet (1987). It consists of 12 self-assessment items across three subscales—support of family, friends, and others. Each item is scored on a 7-point scale, with higher scores indicating a better level of social support. In this study, Cronbach’s alpha coefficient for the scale was 0.951.

#### Subjective well-being

SWB was measured with a Chinese version of the Subjective Well-Being Scale (SWBS). The SWBS consists of two parts: the Satisfaction with Life Scale and the Affective Scale. The Satisfaction with Life Scale developed by Diener et al. (1985), consists of five questions that ask participants to rate how satisfied they are with their lives and how close they are to their ideal lives. The scale is scored on a seven-point scale (1–7 satisfaction levels in ascending order). The internal consistency coefficient of the scale was 0.84. The Affective Scale was based on Affectometer 2 developed by Kammann and Flett (1983). Responses were rated on a 5-point scale ranging from 1 for extremely nonconforming to 5 for very conforming. The scores for the two dimensions were calculated separately. The Cronbach’s alpha coefficient for the Life Satisfaction Scale in this study was 0.915, and the Cronbach’s alpha coefficient for the Affective Scale (positive affect component) was 0.903.

#### Professional commitment

PC was measured with the Chinese version of the Professional Commitment Questionnaire (Zuo and Xi, 2008). It consists of 22 questions and is divided into three commitment dimensions: emotional, sustained, and normative. The questionnaire was scored on a six-point scale, ranging from “very uncommitted” to “very committed.” In this study, the Cronbach’s alpha coefficient for the questionnaire was 0.898.

### Research procedures and statistical methods

Before the implementation of the study, we compiled the selected questionnaires and published them online. Teachers were invited to participate in the online survey, which was available for 2 weeks. We collected 778 valid questionnaires.

Data statistics and analysis were completed using SPSS 23.0 and Amos 23.0, and the analysis techniques included a reliability test, common method deviation test, correlation analysis, and a mediating effect model test.

## Results

### Check for common method bias

The study data were derived from KTs’ self-reports; therefore, there may have been a common method bias. Based on the investigation of confidentiality and the reverse scoring of some items, a Harman single-factor test was used to test data for common method bias (Podsakoff and Organ, 1986). The results showed that seven factors with a characteristic root greater than one were obtained without rotation, and the variance revealed by the first factor was 34.68%, less than the 40% critical standard (Ferguson et al., 2012). Therefore, the results indicated there was no serious common method bias in this study.

### Descriptive statistics

Table 1 shows the average values, SDs, and correlation matrices for each variable. Social support was positively correlated with PA and LS and PC (*p* < 0.01). PA was positively correlated with LS and PC (*p* < 0.01). LS was positively correlated with PC (*p* < 0.01).

**Table 1:** Descriptive statistics and correlation coefficients of the key study variables (*n* = 778).

	1	2	3	4
1 SS	—			
2 PA	0.57^**^	—		
3 LS	0.52^**^	0.73^**^	—	
4 PC	0.30^**^	0.48^**^	0.48^**^	—
*M*	67.90	36.46	22.70	89.88
SD	13.36	6.52	6.70	16.22

SS, social support; PA, positive affect; PC, professional commitment; LS, life satisfaction.

** *p* < 0.01.

### Mediation model test with adjustment

The structural model’s assessment involves the goodness-of-fit (GoF) index, path coefficient, and significance test. The GoF index includes the normed fit index [NFI, >0.8 recommended by Hooper et al. (2008)], root mean square error of approximation (RMSEA, <0.08), GoF index (GFI, >0.9), comparative fit index (CFI, >0.9), and incremental fit index [IFI, >0.9, recommended by Hau et al., 2004]. In this study, we used a latent variable structural model to construct a relationship model between the four variables of social support, PA, LS, and PC to examine the mediating effects among them. The observed variables corresponding to social support were “family,” “friend,” and “other”; the observed variables corresponding to PC were “affective,” “continued,” and “normative.” After correction, the structural equation model fit index was *χ*^2^/df = 2.46 (the remaining fit indexes are shown in Table 2). The results indicate that the model fits well.

**Table 2:** Model fit indexes.

Fit index	RMSEA	CFI	NFI	IFI
Proposed value	<0.08	>0.9	>0.9	>0.9
Estimated value	0.04	0.99	0.99	0.99

RMSEA, root mean square error of approximation; CFI, comparative fit index; NFI, normed fit index; IFI, incremental fit index.

**Figure 2:**
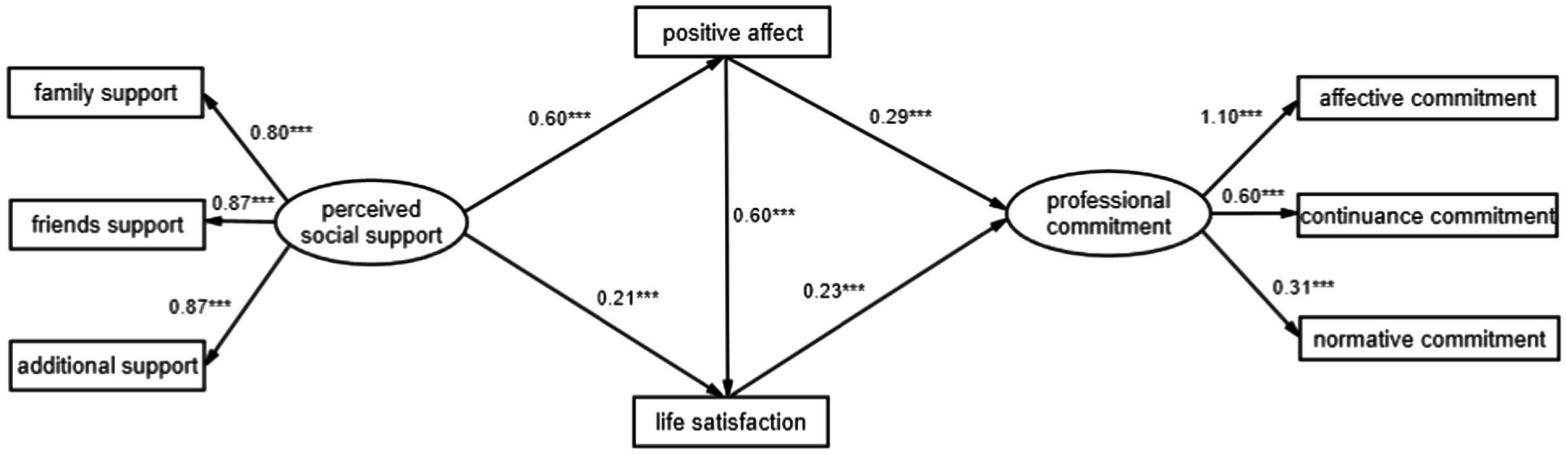
The path relationship between perceived social support, positive affect, and life satisfaction on professional commitment.

The path diagram of the effects of social support, PA, and LS on PC (Figure 2) shows that social support positively predicted PA (*β* = 0.60, *p* < 0.001) and PA positively predicted PC (*β* = 0.29, *p* < 0.001), indicating that social support can indirectly influence PC through the mediation of PA; social support positively predicted LS (*β* = 0.21, *p* < 0.001) and LS positively predicted PC (*β* = 0.23, *p* < 0.001), indicating that social support can have an indirect effect on PC through the mediation of LS. In addition, PA positively predicted LS (*β* = 0.60, *p* < 0.001), suggesting that social support can have an indirect effect on PC through the mediation of PA and LS.

We tested the significance of the mediating effect using bias-corrected percentile Bootstrap analysis (5,000 random samples) with a confidence interval set at 95% (not including 0). The results are shown in Table 3. The mediating effect consisted of indirect effects arising from three pathways: SS → PA → PC; SS → LS → PC; and SS → PA → LS → PC. Within these pathways, SS → PA → PC and SS → LS → PC are parallel multiple mediating effects, and SS → PA → LS → PC indicates the chain multiple mediating effects. Therefore, the indirect effect of KTs’ social support on PC was achieved through compound multiple mediating effects of PA and LS. Thus, H1, H2_,_ and H3 were supported by the results.

**Table 3:** Bootstrap analysis of the significance test of the intermediate model effect.

	Path relationships	Standard indirect effects	Bootstrap (95%CI)	
			Upper limit	Lower limit
Intermediary effect	SS → PA → PC	0.60 × 0.29 = 0.17	0.14	0.20
	SS → LS → PC	0.21 × 0.23 = 0.05	0.03	0.07
	SS → PA → LS → PC	0.60 × 0.60 × 0.23 = 0.08	0.06	0.10
Total intermediation effect		0.6 × 0.29 + 0.21 × 0.23 + 0.60 × 0.60 × 0.23 = 0.3	0.23	0.37

SS, social support; PA, positive affect; PC, professional commitment; LS, life satisfaction.

## Discussion

Previous studies have shown that KTs’ social support is significantly and positively related to PC (Tang et al., 2015; Shi, 2017; Xu et al., 2017). Higher support from leaders results in improved professional commitment from teachers (Kusum and Billingsley, 1998; Singh and Billingsley, 1998). Further, different dimensions of social support are good predictors of SWB (Xu, 2011); emotional support is positively related to LS, and instrumental support is positively related to PA (Jiang, 2020). The higher the SWB of teachers, the higher their work engagement, professional identity, and PC (Wang, 2014; Pi, 2018; Peng, 2021). Our finding—that there were significant positive correlations between KTs’ social support, PA, LS, and professional commitment—is consistent with the results of previous studies (Tang et al., 2015; Zhao, 2016; Shi, 2017; Xu et al., 2017; Jiang, 2020). The factors affecting KTs’ professional commitment are multifaceted, and social support, PA, and LS are important factors. Further, the optimal guarantee of these factors is necessary for teachers to have high professional commitment. When working with children aged 3–6, kindergarten teachers need patience, care and responsibility, and high emotional input and persistence in their work. Only with high recognition and support from families, kindergartens, and society can they maintain positive emotions and high life satisfaction. This is what motivates kindergarten teachers to maintain their PC.

This study found that PA mediated the relationship between social support and professional commitment of KTs, which verified H1, indicating that social support indirectly influences professional commitment through the mediation of PA; LS mediated the relationship between social support and professional commitment of KTs, which verified H2, indicating that social support indirectly influences professional commitment through the mediation of LS. The compound multiple mediating role of PA and LS between KTs’ social support and professional commitment verified H3. The present study is consistent with previous research findings that social support affects PA (Zhao, 2016), LS (Jiang, 2020), job satisfaction, and professional commitment (Daud, 2010; Ferguson et al., 2012; Zhang et al., 2015). It is also consistent with the findings that PA influences LS, and higher LS results in higher SWB (Jiang, 2020). It also supports the findings that higher SWB of teachers is linked with higher job commitment, professional identity, and PC (Wang, 2014; Pi, 2018; Peng, 2021). Meanwhile, this study explores the influence of SWB as a mediating factor in two major dimensions and presents more clearly the compound multiple mediating role of two factors—PA and LS—between social support and professional commitment.

Most KTs are women (Song, 2019). Owing to the different gender roles and social division of labor, particularly among married female teachers, KTs are bound to allocate part of their energy to family life, and the time and energy they devote to their work will be reduced, which will inevitably affect their work engagement and effectiveness. However, recognition and support from family members, relatives, and friends, love and support from kindergarten leaders, and understanding and help from colleagues, enable teachers to gain spiritual comfort at work, maintain positive emotions, and experience pleasure and a sense of accomplishment derived from life and work. They will experience professional value and self-fulfillment in their work, and thus develop emotional commitment and dependence on their profession and be reflected in higher job input and show higher PC. In contrast, as the public attaches importance to preschool children’s development and recognizes the profession of KTs, the social and economic status of KTs will significantly improve. Simultaneously, a series of national preschool education policies and regulations have been introduced to guide the standardized and healthy development of KTs; thus, their working environment has been optimized, and pre-and post-service training mechanisms have improved the levels of professionalism. Teachers’ recognition of their profession has changed, and more teachers are willing to stay at their posts, demonstrating high levels of PC.

## Conclusion

KTs’ social support may not directly predict PC. There are three paths from social support to PC. This social support influences PC through the path of PA and LS, and the compound multiple mediating effect from PA to LS to PC. The provision of social support for KTs should focus on stimulating PA and increasing LS to promote high levels of PC. This will lead to increased love for the job, dedication, and improved quality of teaching.

## Limitations

This study had some limitations, and here, we provide suggestions for addressing them in future research. First, the subjects of this study are from economically developed areas in Guangdong Province, which cannot reflect the general economic level in China. Future studies should expand the sample scope to regions with different economic development levels to increase sample representativeness, thus improving the results’ authenticity and objectivity. Second, the sample is composed of teachers who were willing to participate; their gender, age, years of teaching, education level, and other characteristics are not balanced, which will affect the objectivity and universality of the research results. Future studies should balance these factors as much as possible to reflect the actual composition of kindergarten teachers.

## Data availability statement

The original contributions presented in the study are included in the article/supplementary material, further inquiries can be directed to the corresponding author.

## Author contributions

SC: conceptualization, data curation, writing—original draft, and writing—reviewing and editing. YL: formal analysis and visualization. ZM: writing—reviewing and editing. XC: investigation, data collection, and writing—reviewing. TS: investigation and writing—reviewing. All authors contributed to the article and approved the submitted version.

## Funding

This study was funded by the 2019 Quality Project and Teaching Revolution Program of Zhaoqing University (no. Zlgc201928). 2020 Research Fund Grant Project of Zhaoqing University (no. 202040).

## Conflict of interest

The authors declare that the research was conducted in the absence of any commercial or financial relationships that could be construed as a potential conflict of interest.

## Publisher’s note

All claims expressed in this article are solely those of the authors and do not necessarily represent those of their affiliated organizations, or those of the publisher, the editors and the reviewers. Any product that may be evaluated in this article, or claim that may be made by its manufacturer, is not guaranteed or endorsed by the publisher.
